# Incidence of Tumour Progression and Pseudoprogression in High-Grade Gliomas: a Systematic Review and Meta-Analysis

**DOI:** 10.1007/s00062-017-0584-x

**Published:** 2017-05-02

**Authors:** Abdul W. Abbasi, Henriette E. Westerlaan, Gea A. Holtman, Kamal M. Aden, Peter Jan van Laar, Anouk van der Hoorn

**Affiliations:** 1 University Medical Center Groningen, Department of Radiology, University of Groningen, Hanzeplein 1, 30.001, 9700 RB Groningen, The Netherlands; 2 University Medical Center Groningen, Center for Medical Imaging-North East Netherlands, University of Groningen, Groningen, The Netherlands; 3 University Medical Center Groningen, Department of General Practice, University of Groningen, Groningen, The Netherlands

**Keywords:** Treatment response assessment, Meta-analysis, Pseudoprogression, Incidence, High-grade gliomas

## Abstract

**Background:**

High-grade gliomas are the most common primary brain tumours. Pseudoprogression describes the false appearance of radiation-induced progression on MRI. A distinction should be made from true tumour progression to correctly plan treatment. However, there is wide variation of reported pseudoprogression. We thus aimed to establish the incidence of pseudoprogression and tumour progression in high-grade glioma patients with a systematic review and meta-analysis.

**Methods:**

We searched PubMed, Embase and Web of Science on the incidence of pseudoprogression and tumour progression in adult high-grade glioma patients from 2005, the latest on 8 October 2014. Histology or imaging follow-up was used as reference standard. Extracted data included number of patients with worsening of imaging findings on T1 postcontrast or T2/FLAIR, pseudoprogression and tumour progression. Study quality was assessed. Heterogeneity was tested with *I* ^*2*^. Pooling of the results was done with random models using Metaprop in STATA (StataCorp. Stata Statistical Software. College Station, TX: StataCorp LP).

**Results:**

We identified 73 studies. MRI progression occurred in 2603 patients. Of these, 36% (95% confidence interval [CI] 33–40%) demonstrated pseudoprogression, 60% (95%CI 56–64%) tumour progression and unknown outcome was present in the remaining 4% of the patients (range 1–37%).

**Conclusion:**

This meta-analysis demonstrated for the first time a notably high pooled incidence of pseudoprogression in patients with a form of progression across the available literature. This highlighted the full extent of the problem of the currently conventional MRI-based Response Assessment in Neuro-Oncology (RANO) criteria for treatment evaluation in high-grade gliomas. This underscores the need for more accurate treatment evaluation using advanced imaging to improve diagnostic accuracy and therapeutic approach.

**Electronic supplementary material:**

The online version of this article (doi: 10.1007/s00062-017-0584-x) contains supplementary material, which is available to authorized users. It contains the characteristics of the included studies (supplementary table 1) and a full search strategy (see supplementary search strategy).

## Introduction

Glioblastoma multiforme (GBM) is the deadliest brain cancer, often fatal within a year after diagnosis [1]. This poor prognosis is mainly due to the inevitability of recurrent disease. Imaging is important for accurate treatment evaluation of patients with a glioblastoma. T1-weighted MRI with gadolinium combined with T2/FLAIR is currently the standard imaging technique [2]. However, postcontrast T1 only reflects biological activity of the tumour indirectly, by detecting the breakdown of the blood–brain barrier [3]. T1-weighted MRI does not directly measure tumour size or tumour activity and is non-specific.

Recurrent disease appears as a new contrast-enhancing lesion on T1-weighted MRI or growth of the high T2/FLAIR area. However, a similar presentation may result from treatment effects resulting in the false appearance of disease progression, i.e., pseudoprogression [3–5]. Thus, in the case of progression on imaging, it is necessary to distinguish true tumour progression from pseudoprogression to correctly tailor treatment.

Although recognised as a clinically important problem, there exists a wide variation in the reported incidence of pseudoprogression. Previous studies individually indicating its incidence vary in the range of 3% to over 50% [3, 6]. One of the major limitations of these studies was their small sample sizes. The high variance in the reported incidence of pseudoprogression impedes subsequent treatment decisions.

In order to clarify how often progression occurs, the current meta-analysis systematically reviewed the studies that recorded incidences of pseudoprogression and tumour progression in high-grade glioma patients.

## Methods

### Search Strategy

A systematic review and meta-analysis was performed according to the meta-analysis of observational studies in epidemiology (MOOSE) criteria [7], the preferred reporting items for systematic reviews and meta-analyses (PRISMA) criteria [8], and the assessing the methodological quality of systematic reviews (AMSTAR) guidelines [9].

We systematically searched MEDLINE (PubMed), Embase and Web of Science. Database keywords and text words were used aiming at patients with a high-grade glioma and tumour progression or pseudoprogression, with synonyms for each (see “Appendix” for search strategy). We used both treatment-induced pseudoprogression and radionecrosis in our search strategy, as they belong to a spectrum of radiation-induced injury. We searched the databases from 2005, the time at which temozolomide was included in the standard treatment, till 8 October 2014. No other filters or restrictions were applied. Non-English studies were manually excluded later. Conference proceedings are included in Embase and this thus allowed for the inclusion of grey literature in the meta-analysis. Study selection and data extraction was completed by two authors independently (AA and KA). In the case of inconsistencies, a third author was consulted (AH or HW).

### Selection Criteria

Inclusion criteria were studies having a consecutive or random selection of adult patients diagnosed with a high-grade glioma following standard care of treatment with first-line concomitant chemoradiotherapy with temozolomide, followed by adjuvant temozolomide. Surgical resection was not mandatory for inclusion, as some patients did not receive surgical resection due to contraindications, such as comorbidity. Histological confirmation, imaging follow-up, or a combination of the two had to be used as a reference standard to identify pseudoprogression or true tumour progression in patients with a form of imaging progression. In the cases where a definitive diagnosis could not be established, progression was classified as unknown.

Exclusion criteria included patients with recurrent disease. Any patient group or study that did not follow the characteristics described in the inclusion criteria above, like a group of mixed high- and low-grade gliomas, were also excluded. Studies exploring gliomas of the cranial nerves and spine were excluded. Finally, use of new therapies was also excluded due to our interest in the standard patient group.

### Study Selection, Data Extraction and Quality Assessment

Main data extracted were the number of patients with any form of progression on MRI, the number of patients with pseudoprogression and the number of patients with tumour progression. General study characteristics were also extracted. These included study design, total number of patients, percentage of males, patients’ age with range, reference standard (histology and/or follow-up), definition of tumour progression and pseudoprogression, image protocol, and interval between end of therapy and progression. Quality of included studies was assessed with the NIH Quality Assessment Tool by two authors independently [10]. The NIH Quality Assessment tool was divided into four domains. These included the general study setup (questions 1 and 14), the patient selection domain (questions 2–5), the follow-up domain (question 7) and the reference standard domain (questions 11 and 13). Questions 6, 7, 10 and 11 were excluded. These questions were considered non applicable, as the exposure that was referred to was similar for all patients as we included only patients after standard treatment.

### Statistical Analysis

Meta-analysis was performed using data extracted from each study. SPSS version 23 (IBM Inc., Armonk, NY, USA) was employed to calculate the general patient characteristics. The incidence of patients with tumour progression, pseudoprogression or unknown progression was calculated per study. Subsequently, pooled results of tumour progression and pseudoprogression incidences were calculated using Metaprop in STATA/SE 12.1 (College station, TX, USA) [11]. Studies were weighted according to their variance and the sample size. The I^2^ test was used to calculate the heterogeneity of the included studies. As this demonstrated a heterogeneous study set, a random effects model was utilised to calculated pooled estimates.

### Role of Funding Source

The funder of the study had no role in study design, data collection, data analysis, data interpretation or report writing. The corresponding author had full access to all data in the study and had final responsibility for the decision to submit for publication.

## Results

### General Description of Selection and Included Studies

**Fig. 1:**
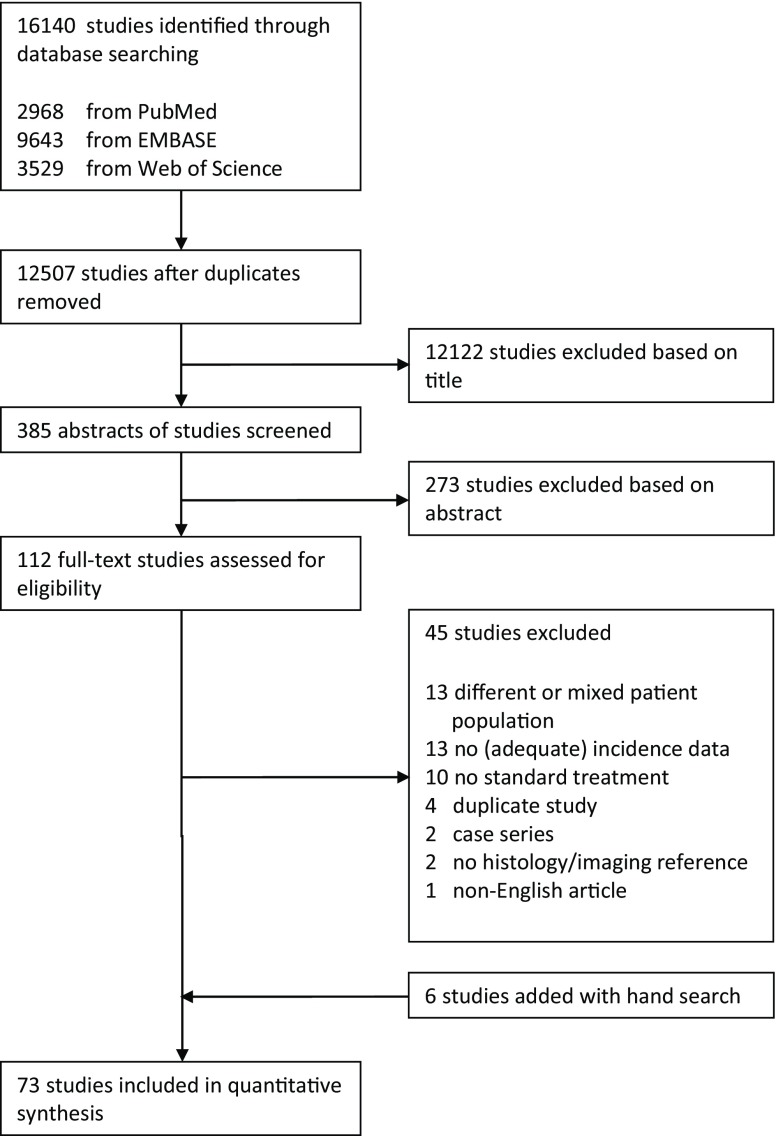
Flowchart demonstrating the inclusion and exclusion of studies

We started with 12,507 unduplicated studies. Among the 112 articles that were reviewed in depth, a total of 67 studies met the eligibility criteria and were included in the meta-analysis [12–78]. Additionally, six articles [79–84] were identified with a hand search, leading to a total of 73 included studies (Fig. 1 and appendix for table 1). Fifteen abstracts were included (21%) [12, 25, 31, 32, 36, 38, 45, 51, 53, 58, 60, 66, 71, 73, 76].

The included studies concerned 3781 patients with a mean age of 54 years. Of all patients, 57% were male. The studies included 89% WHO grade IV astrocytomas (glioblastomas), 7% WHO grade III astrocytomas (anaplastic astrocytomas) and no specification in 4% of the patients. In the majority of the patients (75%), imaging follow-up was used as the reference standard. Histology was utilised in 20% of the patients. A combination of both histopathology and imaging follow-up was used in 2% of the patients (see supplementary table 1 for details including the definitions of tumour progression and pseudoprogression). Clinical follow-up alone was used in 3 patients (0.09%), while it was unknown for two studies with a total of 94 patients (2.7%) [45, 58]. Sufficient data was provided in 40 studies to calculate the average follow-up period after initial progression on imaging, with a mean follow-up of 14 months (range 1–67 months).

### Quality of Included Studies

**Fig. 2:**
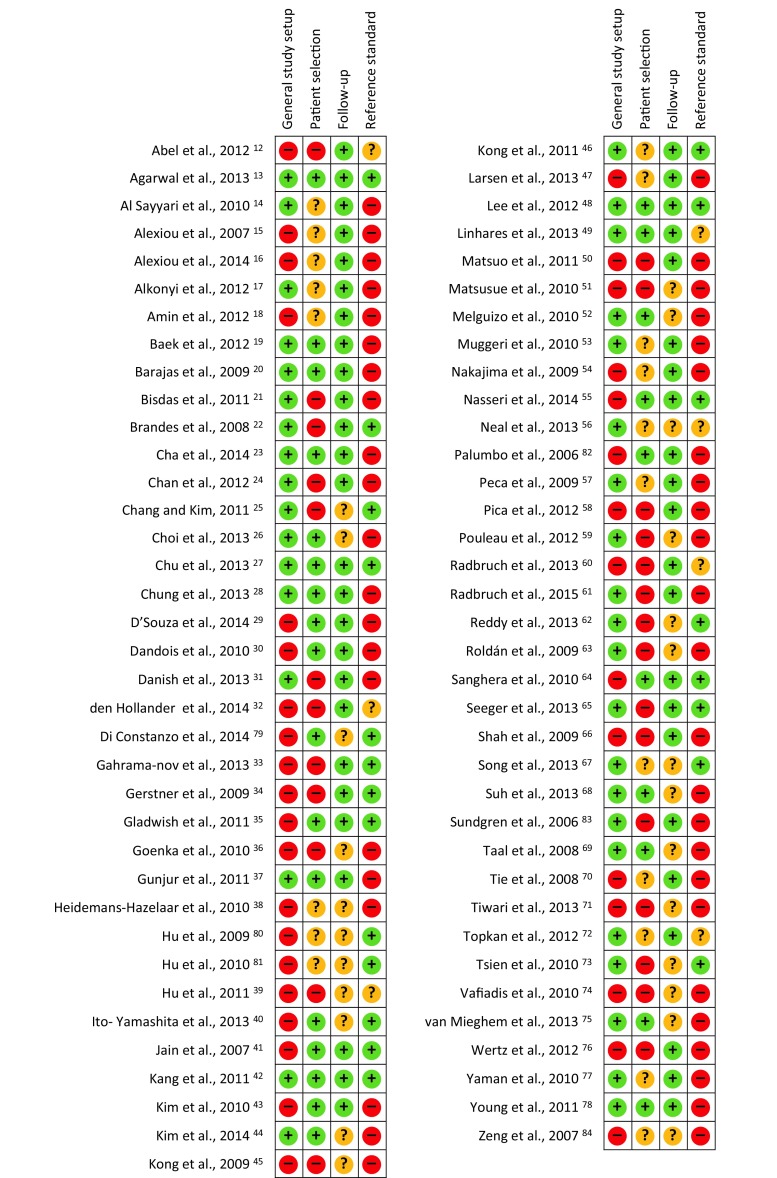
Quality assessment of included studies. The risk of bias in four different domains and concerns about applicability are shown for the included studies. High risk (*red* −), unclear risk (*yellow* ?) and low risk (*green* +)

A summary of the methodological quality assessment of the included articles is presented in Fig. 2. For the general study setup, a moderate risk was identified. No statistical analysis for potential confounding variables like follow-up duration, MGMT status or used reference standard was performed in 36 (49%) studies and they were thus classified as high risk [12, 15, 16, 18, 29, 30, 32–36, 38–41, 43, 45, 47, 50, 51, 54, 55, 57, 59, 64, 66, 70, 71, 73, 76, 79–82, 84]. The remaining 37 (51%) studies showed no risk for these questions [13, 14, 17, 19–28, 31, 37, 42, 44, 46, 48, 49, 52, 53, 56, 57, 59, 61–63, 65, 67–69, 72, 73, 75, 77, 78, 83].

In the patient selection domain, all articles were classified as high-risk due to the fact that no article had mentioned sample size calculation or power discussion, nor variance or estimate of effect size (question 5 of NIH assessment tool). As the influence of this item on the quality was considered negligible, the patient domain was approached after removing question 5. This resulted in a much lower risk classification with a total of 27 (37%) high-risk studies. Lacking information pertaining to the number of WHO III and IV tumours [65], as well as age; [21], sex [24, 34, 45, 73], or the latter two [31–33, 39, 50, 58, 60, 61, 71, 76] or three items [12, 25, 36, 74], resulted in a high-risk classification in 20 studies. A participation rate below 50% resulted in an additional 7 high risk studies [22, 51, 59, 62, 63, 66, 83]. Unclear risk was seen in 19 (26%) studies, as the participation rate was not reported [14–18, 38, 46, 53, 54, 56, 57, 67, 70, 72, 77, 80, 81, 84]. Low risk was present in the remaining 27 studies (37%) [13, 19, 20, 23, 26–30, 35, 37, 40–44, 48, 49, 52, 55, 64, 68, 69, 75, 78, 79, 82].

In the follow-up domain, the average quality assessment was good. A total of 25 studies (34%) were unclear about the follow-up duration [25, 26, 36, 38–40, 44, 45, 51, 52, 56, 59, 62, 63, 67–69, 71, 73–75, 79–81, 83], while the remaining 48 (66%) were low risk, utilising a sufficient time window to classify patients as presenting with either tumour progression or treatment-related progression [12–24, 27–35, 37, 41–43, 46–50, 53–55, 57, 58, 60, 61, 63–66, 70, 72, 76–78, 82, 83].

For the reference standard domain, the overall assessment was deemed a moderate risk. Overall, 45 (62%) studies were high risk. A total of 8 did not report on reference standard in sufficient detail [45, 50, 53, 54, 57, 58, 71, 82], 23 studies did not apply the same reference (histology and/or imaging) to all subjects [14, 17–21, 23, 29, 37, 38, 43, 44, 47, 51, 52, 66, 68, 69, 74, 77, 78, 83, 84] and 6 studies did not contain the latter two items [15, 16, 26, 30, 36, 70]. A lost to follow-up rate >20% resulted in high-risk classification of another 8 studies [24, 28, 31, 59, 61, 63, 75, 76]. The lost to follow-up rate was unclear in 7 studies (10%), resulting in an unclear risk [12, 32, 39, 49, 56, 60, 72]. The remaining 21 (29%) studies were considered low risk with respect to these items [13, 22, 25, 27, 33–35, 40–42, 46, 48, 55, 62, 64, 65, 67, 73, 79–81].

### Heterogeneity

The *I* ^*2*^ index demonstrated that the included studies were heterogeneous for the incidence of pseudoprogression (*p* < 0.01, *I* ^*2*^ = 79%) and for the incidence of tumour progression (*p* < 0.01, *I* ^*2*^ = 82%). To account for this heterogeneity, a random effects model was utilised for the analyses of pooled results.

### Incidence of Pseudoprogression and Tumour Progression

**Fig. 3:**
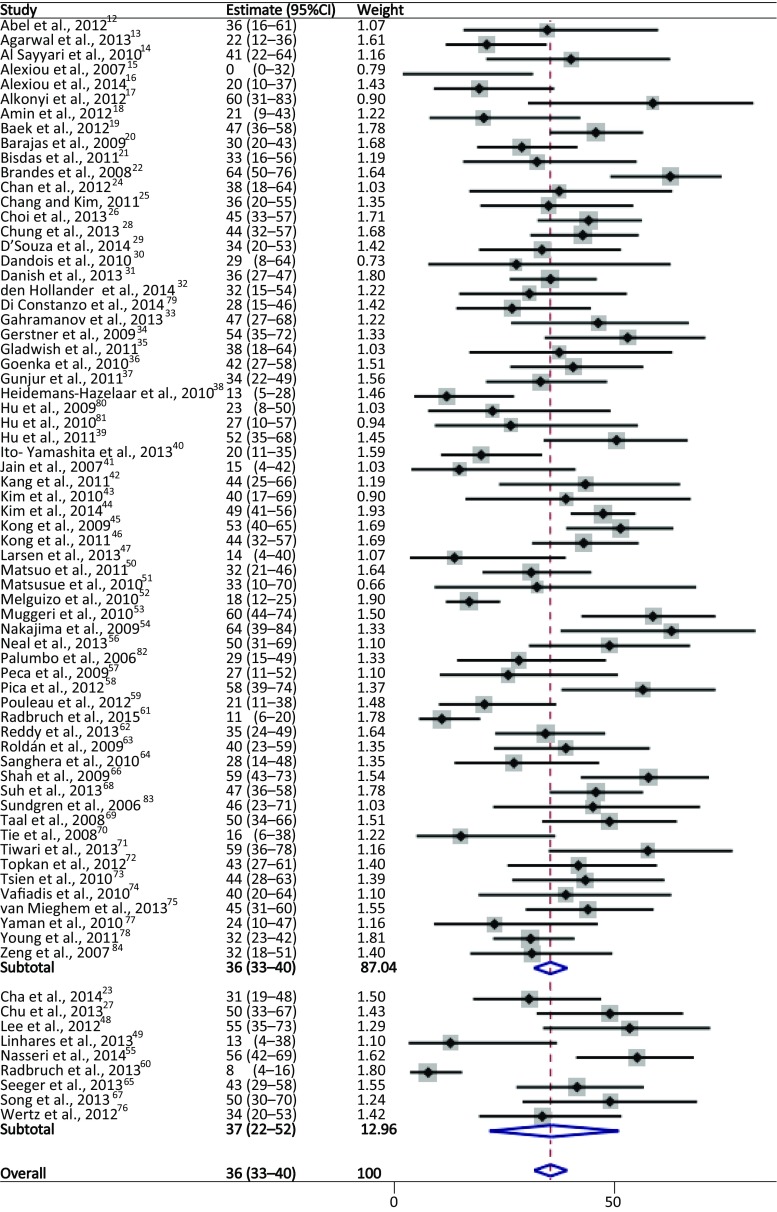
Forest plot of pooled incidences for treatment induced pseudoprogression. *Squares* represent the weighted estimate with the solid line indicating the 95% CI. The *dashed line* represents the group estimate

Of the total number of included patients in all studies, 2603 patients displayed some form of worsening of imaging findings, demonstrating increased or new enhancement on postcontrast T1 or progression of high signal on T2/FLAIR imaging according to the RANO criteria (Fig. 3). Of these patients with progression, 36% (95%CI 33–40%) displayed pseudoprogression due to treatment effects. A total of 60% (95%CI 56–64%) of the patients with progression were diagnosed with true tumour progression. The remaining 4% of patients spread over 12 studies showed an unknown outcome (range 1–37%). In a subset of 9 studies (*N* = 295), where the use of the RANO criteria to identify progression was specifically stated [23, 27, 48, 49, 55, 60, 65, 67, 76], the pooled results were similar, with 37% (95%CI 22–52%) of the patients showing pseudoprogression. Furthermore, heterogeneity testing results for the RANO group and the other studies were similar (*p* = 1.00), justifying the pooling of both groups. Comparing the abstract only studies with the others full-text studies showed no clear difference looking at the forest plot.

The interval between the end of concomitant chemoradiotherapy and the time of tumour progression or pseudoprogression on imaging was available for 11 studies, including 265 patients with tumour progression and 204 patients with pseudoprogression. The mean interval was 10.5 months (range 1.7–37.6 months) for tumour progression. For pseudoprogression the interval was 13.0 months on average (range 1.2–40.0 months).

## Discussion

Worsening of imaging findings on postcontrast T1 or high T2/FLAIR MRI can be attributed to tumour progression or pseudoprogression, but the distribution of both was highly uncertain prior to this study. Our meta-analysis has shown that across the available literature, pseudoprogression occurred, on average, in 36% of the patients with a high-grade glioma, while tumour progression occurs in 60%.

The finding that 36% of the patients presenting with progression is due to pseudoprogression confirms what many experts in the field suspect; pseudoprogression is a frequently occurring entity. However, it is above rates stated commonly in the literature. For instance, an elaborate review indicated that pseudoprogression can occur in up to 20% of patients treated with standard temozolomide chemoradiotherapy [3], with a reported range of 3–24%. Previous studies providing incidence data have based their findings on a limited number of studies while utilising a non-systematic search approach. Considering the large amount of available data, it is also hard to do it differently, unless a full meta-analysis is conducted. Our meta-analysis is thus the first study to combine the data from all the available literature to estimate an incidence reflecting the full extent of the available literature. This is also the first study to specifically calculate pseudoprogression incidence consistently for all studies only including patients with some form of imaging progression. Including patients without any imaging progression would have resulted in lower and hard-to-interpret numbers which cannot be compared with other studies easily.

The occurrence of neurological deficits due to tumour progression or recurrence on MR imaging has been reported to be accompanied by the presence of oedema [85], which was already demonstrated in 1979 [86]. In that study, it was noted that 28% of the patients spontaneously improved without a change in prescribed therapy. This is now recognised as being due to pseudoprogression. The pathophysiology of pseudoprogression remains unclear. Demyelination was discussed as a probable factor [87]. A more commonly used explanation is the increased capillary permeability induced by radiotherapy [88]. In conjunction with its disruption, upregulation of signalling proteins also affects the permeability of the blood–brain barrier [89]. This meta-analysis, to our knowledge, collates separate observations of all previous literature for the first time, to provide a more complete overview of progression in the context of high-grade glioma treatment.

By clarifying the full extent of the known limitations, this meta-analysis will enable clinicians to more carefully interpret posttreatment conventional MRI imaging in patients showing progression. Supplementary research is needed to improve the differentiation between true tumour progression and pseudoprogression. This could be achieved using more advanced MRI and/or PET imaging to visualise the biological activity of tissue. Firstly, this improved knowledge is needed for deciding which patients do not benefit from the treatment given. The therapy and its side-effects can be discontinued and a switch to a second-line treatment could be considered. Secondly, new immunotherapies or antiangiogenic medications present new problems in the judgment of progression on anatomical MRI [90]. Thus, further research on functional imaging for treatment follow-up is paramount.

This review was limited due to the nature of the available literature. Most importantly, defining tumour progression and pseudoprogression is challenging. This is reflected in the variability in the definitions used in the included studies (see supplementary table 1). However, all studies used histology or imaging follow-up that needed to show some form of stabilisation or improvement in cases with pseudoprogression, which are adequate definitions. Some of the abstract-only studies that were included to prevent publication bias provided no clear definition. This was reflected in the moderate quality assessed with the NIH quality assessment tool. The retrospective nature of some included studies was also a limitation affecting the NIH quality assessment scores. None of the studies reported on power or sample sizes. This is because observational studies are often exploratory in nature. However, the risk of an insufficient sample size is overcome by the benefits of this meta-analysis combining all studies.

In conclusion, this meta-analysis showed that, across the available literature weighted by importance, pseudoprogression occurred frequently (36%) in patients with a high-grade glioma following standard chemoradiotherapy. Tumour progression occurred in 60% of the patients with some form of imaging progression. This meta-analysis thus showed the full extent of the problem in differentiating pseudoprogression from tumour progression, helping pave the way towards more research to improve imaging methods for reliable treatment decision making.

### Caption Electronic Supplementary Material

Table 1 Characteristics of included studies. Characteristics of 73 included studies. *AMT* α-methyl-L-tryptophan, *C* carbon, *CCRT* concomitant chemoradiotherapy, *CT* computed tomography; *d* days, *DCE* dynamic contrast-enhanced perfusion, *DMSA* dimercaptosuccinic acid, *DSC* dynamic susceptibility contrast perfusion, *DTI* diffusion tensor imaging, *DWI* diffusion weighted imaging, *FLAIR* fluid attenuated inversion recovery, *IQR* interquartile range, *MET* methionine, *Mo* months, *MRI* magnetic resonance imaging, *MRS* magnetic resonance spectroscopy, *N* number, *PET* positron emission tomography, *Pros* prospective, *PWI* perfusion weighted imaging, *Retro* retrospective, *SD* standard deviation, *SPECT* single positron emission computed tomography, *SWI* susceptibility weighted imaging, *T* Tesla, *T1C* T1 post contrast; *Tc* technetium; *TP* tumour progression, *trP* treatment induced progression, *WHO* World Health Organisation, *wk* weeks, *y* years

## Appendix

### Search Strategy

#### Search Strategy MEDLINE Via PubMed

(("2005/01/01"[Date - Publication] :"3000"[Date - Publication]) AND((gliomas[MeSH]) OR (brain neoplasm[MeSH]) OR(glioma*[Tiab]) OR(glioblastom*[Tiab]) OR(“brain neoplasm”[Tiab]) OR(“brain neoplasms”[Tiab]) OR(“brain tumour”[Tiab]) OR(“brain tumours”[Tiab]) OR(“brain tumor”[Tiab]) OR(“brain tumors”[Tiab]) OR(“brain cancer”[Tiab]) OR(“brain malignancies”[Tiab]) OR(“brain malignancy”[Tiab]) OR(“cerebral neoplasm”[Tiab]) OR(“cerebral neoplasms”[Tiab]) OR(“cerebral tumour”[Tiab]) OR(“cerebral tumours”[Tiab]) OR(“cerebral tumor”[Tiab]) OR(“cerebral tumors”[Tiab]) OR(“cerebral cancer”[Tiab]) OR(“cerebral malignancy”[Tiab]) OR(“cerebral malignancies”[Tiab])) AND((“disease progression”[MeSH]) OR(pseudoprogression[Tiab]) OR(radionecros*[Tiab]) OR(“tumor progression”[Tiab]) OR(“tumour progression”[Tiab]) OR(“radiation necrosis”[Tiab]) OR(“pseudo progression”[Tiab]) OR(pseudoprogression[Tiab]) OR(pseudo-progression[Tiab]) OR(radio-necrosis[Tiab])))

#### Search Strategy EMBASE Via EMBASE

([2005-2014]/py) AND ('glioma'/exp ORbrain cancer'/exp OR glioma:ab,ti ORglioblastoma:ab,ti ORbrain neoplasma':ab,ti ORbrain neoplasmas':ab,ti OR‘brain cancer’:ab,ti OR‘brain malignancy’:ab,ti OR‘brain malignancies’:ab,ti ORbrain tumor':ab,ti ORbrain tumour':ab,ti ORbrain tumors':ab,ti ORbrain tumours':ab,ti ORcerebral neoplasma':ab,ti ORcerebral neoplasmas':ab,ti ORcerebral tumor':ab,ti ORcerebral tumour':ab,ti ORcerebral tumors':ab,ti ORcerebral tumours':ab,ti OR‘cerebral cancer’:ab,ti OR‘cerebral malignancy’:ab,ti OR‘cerebral malignancies’:ab,ti) AND('tumor growth'/exp ORradiation necrosis'/exp ORcancer growth'/exp ORdisease progression':ab,ti ORpseudoprogression':ab,ti OR‘pseudo-progression’:ab,ti OR ‘radio-necrosis’:ab,ti ORradionecros*:ab,ti ORtumor progression':ab,ti ORtumour progression':ab,ti ORradiation necrosis':ab,ti ORpseudoprogression':ab,ti)

#### Search Strategy Web of Science Via Web of Knowledge

TS=(glioma* OR "brain neoplasma" ORbrain neoplasm” OR "glioblastoma" ORbrain neoplasm" OR“brain neoplasma” ORbrain tumor" OR “brain tumour” ORbrain tumours” OR “brain tumors” ORbrain cancer” ORbrain malignancy” ORbrain malignancies” ORcerebral neoplasm" ORcerebral neoplasma” ORcerebral tumor" ORcerebral tumour” ORcerebral tumors” ORcerebral tumours” ORcerebral cancer” ORcerebral malignancies” ORcerebral malignancy”) ANDTS=("disease progression" ORpseudoprogression" ORradionecrosis" ORtumor progression" ORtumour progression" ORradiation necrosis" ORpseudo progression" ORpseudo-progression” ORradio-necrosis”)
