# Supplementary material for: Incidence of Tumour Progression and Pseudoprogression in High-Grade Gliomas: a Systematic Review and Meta-Analysis
Source: Clin Neuroradiol. 2017 May 2;28(3):401–11. doi: 10.1007/s00062-017-0584-x (PMC6105173; doi:10.1007/s00062-017-0584-x)
Supplement: Supplementary file 1 — Table 1 Characteristics of included studies. Characteristics of 73 included studies. AMT α-methyl-L-tryptophan, C carbon, CCRT concomitant chemoradiotherapy, CT computed tomography; d days, DCE dynamic contrast-enhanced perfusion, DMSA dimercaptosuccinic acid, DSC dynamic susceptibility contrast perfusion, DTI diffusion tensor imaging, DWI diffusion weighted imaging, FLAIR fluid attenuated inversion recovery, IQR interquartile range, MET methionine, Mo months, MRI magnetic resonance imaging, MRS magnetic resonance spectroscopy, N number, PET positron emission tomography, Pros prospective, PWI perfusion weighted imaging, Retro retrospective, SD standard deviation, SPECT single positron emission computed tomography, SWI susceptibility weighted imaging, T Tesla, T1C T1 post contrast; Tc technetium; TP tumour progression, trP treatment induced progression, WHO World Health Organisation, wk weeks, y years [file 62_2017_584_MOESM1_ESM.docx]

**TABLE 1 – Characteristics of included studies**

| **Study** | **Study design** | ***N*** | **%**  **male** | **Age mean ± SD**  **(range)** | **Tumour Histology** | **Reference standard** | **Imaging criteria progression, tumour progression and treatment induced progression** | **Follow-**  **up**  **period** | ***N*  progres-sion (in-crease or new en-hance-ment** | ***N***  **tu-mour pro-gres-sion** | ***N* treat-ment pro-gres-sion** | ***N***  **un-clear pro-gres-sion** | **Interval**  **between time of progress-sion and end CCRT** |
| --- | --- | --- | --- | --- | --- | --- | --- | --- | --- | --- | --- | --- | --- |
| Abel et al., 2012 (abstract) ^12^ | Retro | 14 | - | - | WHO III+IV: 14 | Imaging follow-up (*N*=14) | Progression is worsening contrast enhancement within 6 mo postCCRT. Based upon subsequent MRI progression or stabilization, patients were divided into TP or trP. | 6∙8 mo | 14 | 9 | 5 | 0 | TP/TrP: <6 mo postCCRT |
| Agarwal et al., 2013 ^13^ | Retro | 163 | 18 | 57 | WHO III: 6  WHO IV: 40 | Imaging follow up (*N*=46) | Progression was defined according to the MacDonald criteria. | 412 d | 46 | 36 | 10 | 0 | TP: 51 (11-89) d  TrP: 36 (11-87) d postCCRT |
| Al Sayyari et al., 2010 ^14^ | Pros | 17 | 47 | 53 ± 17∙4  (29-92) | WHO III: 6  WHO IV: 11 | Histology (*N*=5), imaging follow-up (*N*=12) | TP was defined as a steady increase in enhancement and mass effect despite steroid therapy, in combination  with deteriorating neurologic symptoms. TrP was stable imaging or resolving regions of enhancement for ≥6 mo, accompanied by neurologic improvement. | 7∙9 (3-26) mo post-CCRT | 17 | 10 | 7 | 0 | - |
| Alexiou et al., 2007 ^15^ | Pros | 8 | 75 | 52  (31-63) | WHO III: 4  WHO IV: 4 | Histology (*N*=4), imaging follow-up (*N*=4) | - | 12 mo | 8 | 8 | 0 | 0 | TP: 9∙8 ± 15∙8) mo postCCRT |
| Alexiou et al., 2014 ^16^ | Pros | 30 | 70 | 62 ± 11∙1 | WHO III: 3  WHO IV: 27 | Histology (*N*=2), imaging follow-up (*N*=28) | - | 6 mo | 30 | 24 | 6 | 0 | TP/TrP: 12 mo (range 3-24) post CCRT |
| Alkonyi et al., 2012 ^17^ | - | 10 | 82 | 45 ± 10∙6  (30-61) | WHO III: 4  WHO IV: 6 | Histology (*N*=7), imaging follow-up (*N*=3) | TP on histology showed evidence of tumour at the same or higher grade, or if there was clinical deterioration plus growth of an enhancing lesion on MRI during a 1-y follow-up after the 11C-AMT PET scan. TrP was defined as histology verified presence of radiation injury without evidence of definite tumour, or if the patient remained clinically stable or improved, with a stable or diminishing enhancement or high T2 or FLAIR area on MRI during a 1-y follow-up after the 11C-AMT PET. | ≥1 year | 10 | 4 | 6 | 0 | TP: 26 ± 9 mo  TrP: 40 ± 13 mo postCCRT |
| Amin et al., 2012 ^18^ | - | 19 | 54 | 55  17-70 | WHO III: 12 WHO IV: 7 | Histology (*N*=5), imaging follow-up (*N*=19) | TP is continued progression or partial regression after therapy on follow-up imaging or biopsy. TrP was affirmed negativity on follow-up unaccompanied by clinical worsening. | 12 mo | 19 | 15 | 4 | 0 | - |
| Baek et al., 2012 ^19^ | Retro | 128 | 58 | 49  (± 13) | WHO IV: 128 | Histology (*N*=22), imaging follow-up (*N*=57) | TP is a steady increase on MRI with contrast. TrP is neurologic compromise, or resolution of previously enhancement lesions at regular follow-up MRI performed 2–3 mo later. | 2-3 mo | 79 | 42 | 37 | 0 | - |
| Barajas et al., 2009 ^20^ | Retro | 57 | 58 | 54  (± 10∙2) | WHO IV: 57 | Histology (*N*=55), imaging follow-up (*N*=2) | Histology was used as reference or decreasing size of the enhancement on follow-up for indicating TrP | 32∙7 mo | 57 | 40 | 17 | 0 | TP: 16∙2 ± 10∙7 mo  TrP:  22∙9 ± 12∙3 mo postCCRT |
| Bisdas et al., 2011 ^21^ | Pros | 18 | 56 | - | WHO III: 5  WHO IV: 7 | Histology (*N*=5), imaging follow-up (*N*=13) | TP was a steady increase in enhancement and mass effect despite steroid treatment. TrP was stable or resolving enhancement for ≥ 6 mo, accompanied by neurologic improvement. | 9-12 mo | 18 | 12 | 6 | 0 | - |
| Brandes et al., 2008 ^22^ | Pros | 103 | 66 | 52  (20-73) | WHO IV: 103 | Imaging follow-up (*N*=103) | TrP was if the lesion was stable or had improved; otherwise they were registered as early TP using the MacDonald criteria | 18∙9 mo | 50 | 18 | 32 | 0 | TP: 2∙7 mo  TrP: 17∙7 mo postCCRT |
| Cha et al., 2014 ^23^ | Retro | 35 | 51 | 49 (24-70) | WHO IV: 35 | Histology (*N*=3), imaging follow-up (*N*=32) | Imaging follow-up used the RANO criteria. | 556 ± 288 d | 35 | 24 | 11 | 0 | TP/TrP: 123 ± 18 d post surgery |
| Chan et al., 2012 ^24^ | Retro | 28 | - | 48 (16-71) | WHO IV: 28 | Imaging follow-up (*N*=28) | Progression as defined by an independent neuroradiologist according to the Macdonald criteria. | > 6 mo | 13 | 8 | 5 | 0 | - |
| Chang and Kim, 2011 (abstract) ^25^ | - | 55 | - | - | WHO III + IV: 55 | Imaging follow-up (*N*=55) | Progression was defined as radiologic progression within ≤4 wk after CCRT. TP showed further progression and trP showed improvement on follow-up. | - | 25 | 16 | 9 | 0 | TP/TrP: <4 wk postCCRT |
| Choi et al., 2013 ^26^ | Retro | 117 | 60 | 49  (22-79) | WHO IV: 117 | Histology (*N*=19), imaging follow-up (*N*=43) | - | - | 62 | 34 | 28 | 0 | TP/TrP: <4 wk postCCRT |
| Chu et al., 2013 ^27^ | Retro | 52 | 53 | 51  (25-72) | WHO IV: 53 | Imaging follow-up (*N*=52) | Progression was defined according to the RANO criteria | 6 mo | 30 | 15 | 15 | 0 | TP/TrP: 23 d (1-35) postCCRT |
| Chung et al., 2013 ^28^ | Retro | 256 | 60 | 49  (22-79) | WHO IV: 57 | Histology (*N*=57) | TP pathology included cellular sheets and/or nests of atypical cells, often with mitotic figures. If necrosis  was present, it was a minor component of the cellular tumour. The finding of fewer atypical cells, in a linear infiltrative configuration in the parenchyma without prominent reactive changes, was also classified as tumour. TrP was: geographic coagulative necrosis without pseudo-palisading, vascular necrosis, vascular hyalinization, reactive vascular changes, dystrophic calcification, perivascular chronic inflammation, and gliosis with atypia. A sample with a mixture of necrosis and tumour was classified as tumour. | 40 mo postCCRT | 57 | 32 | 25 | 0 | TP: 9∙3 ± 3∙8 mo  TrP: 10∙8 ± 5 mo |
| D’Souza et al., 2014 ^29^ | Pros | 29 | 74 | 43  (18-61) | WHO III:16  WHO IV: 13 | Histology (*N*=22), imaging follow-up (*N*=7) | TrP on imaging showed evidence of lesion stability or regression on follow-up MRI/PET and stable neurological symptoms. | 14 (6-28) mo postCCRT | 29 | 19 | 10 | 0 | - |
| Dandois et al., 2010 ^30^ | Retro | 7 | 57 | 51  (25-74) | WHO III: 1  WHO IV: 6 | Histology (*N*=2), PET imaging (*N*=4), clinical follow-up (*N*=1) | - | 13 (3-40) mo | 7 | 5 | 2 | 0 | - |
| Danish et al., 2013 (abstract) ^31^ | Retro | 131 | - | - | WHO IV: 131 | Imaging follow-up (*N*=131) | Progression was defined as increasing enhancement ≤3 mo postCCRT. TP was defined as continued progression beyond the initial 3 month window. TrP was defined as progression that subsequently stabilized/ improved ≤6 mo postCCRT without further salvage therapy. | 12 mo | 85 | 30 | 31 | 24 | - |
| den Hollander et al., 2014 (abstract) ^32^ | Pros | 30 | - | - | WHO IV:30 | Imaging follow-up (*N*=19) | Progression was classified using the Macdonald criteria. TrP was progressive disease on MRI after CCRT, with stabilization or improvement of enhancing lesions after 3 cycles of adjuvant TMZ | 4 weeks after RT | 19 | 6 | 6 | 7 | - |
| Di Constanzo et al., 2014 ^79^ | Pros | 29 | 62 | 63  (38-74) | WHO IV: 29 | Imaging follow-up (*N*=29) | TP is steady growth or marked enlargement of enhancement and mass effect, despite steroid therapy, on ≥2 follow-up MRI. TrP is stable-appearing or regressing regions of enhancement on at ≥4 follow-up MRI scans. | - | 29 | 21 | 8 | 0 | - |
| Gahrama-nov et al., 2013 ^33^ | Pros | 19 | - | - | WHO IV: 19 | Imaging follow-up (*N*=19) | Progression was based on the high rCBV with ferumoxytol and high rCBV with gadoteridol. For trP both needed to be low. Images was interpreted by a blinded neuroradiologist | 314 (95%CI 156-591) d | 19 | 9 | 9 | 1 | 6 mo |
| Gerstner et al., 2009 ^34^ | Retro | 45 | - | 56 (IQR 48-64) | WHO IV: 45 | Imaging follow-up (*N*=45) | Progression was defined according to the MacDonald criteria | >6 months | 24 | 11 | 13 | 0 | TP/TrP: 25 d postCCRT |
| Gladwish et al., 2011 ^35^ | Pros | 25 | 60 | 56 (46-68) | WHO IV: 25 | Imaging follow-up (*N*=25) | TP was any radiological progression from baseline to one month post-RT as defined by a radiation oncologist. TrP was progression on imaging but the patient showed clinically stable disease for at ≥6 mo post-RT without a change in the adjuvant chemotherapy regimen. | 26∙3 (13∙3-37∙7) mo | 13 | 8 | 5 | 0 | TP/TrP: 7∙5 mo |
| Goenka et al., 2010 (abstract) ^36^ | Pros | 32 | - | - | WHO III+IV: 32 | Histology and/or imaging follow-up (*N*=32) | - | - | 36 | 21 | 15 | 0 | - |
| Gunjur et al., 2011 ^37^ | - | 68 | 66 | 58  (23-75) | WHO IV: 68 | Histology (*N*=7), imaging follow-up (*N*=61) | TP was defined as continued progression on the 3 months MRI following the first postCCRT MRI. TrP was defined as radiological progression on the first postCCRT scan, with further imaging within 3 months  being stable or improving. | 11∙6 mo | 41 | 27 | 14 | 0 | TP: 10∙9 mo  TrP: 3∙1 mo postCCRT |
| Heidemans-Hazelaar et al., 2010 (abstract) ^38^ | Retro | 32 | 85 | 48  (31-62) | WHO IV: 32 | Histology or imaging follow-up (*N*=32) | TrP was defined as absence of TP at a second biopsy or reoperation, or no further progression or spontaneous improvement on follow-up MRI without new anti-tumour therapy nor increase in dexamethasone dose. | - | 32 | 28 | 4 | 0 | - |
| Hu et al., 2009 ^80^ | Pros | 13 | 85 | 48  (31-62) | WHO III: 4  WHO IV: 9 | Histology (*N*=13) | TP on histology included sheets and/or nests of atypical cells often with mitotic figures. Necrosis involved cellular tumour rather than parenchyma. TrP included paucicellularity, lack of numerous atypical cells, lack of mitotic figures, and reactive cells, including gemistocytes. Necrosis, if present, involved parenchyma rather than cellular tumour. Samples containing a mixture of both tumour and treatment effect were classified as tumour, regardless of the percentage of tumour bulk. | - | 13 | 6 | 3 | 4 | TP/TrP: 19∙7 mo postCCRT |
| Hu et al., 2010 ^81^ | Pros | 11 | 91 | 47 | WHO III: 3  WHO IV: 8 | Histology (*N*=11) | See definition of Hu et al., 2009 | - | 11 | 5 | 3 | 3 | - |
| Hu et al., 2011 ^39^ | Pros | 31 | - | - | WHO IV: 31 | Imaging follow-up (*N*=31) | TP were enhancing regions that significantly increased in size. TrP was enhancement that remained stable or decreased in size. | - | 31 | 15 | 16 | 0 | - |
| Ito- Yamashita et al., 2013 ^40^ | Retro | 44 | 68 | 58  (30-84) | WHO III: 18  WHO IV: 26 | Imaging follow-up (*N*=44) | Progression was based on the RECIST criteria | - | 44 | 35 | 9 | 0 | - |
| Jain et al., 2007 ^41^ | Pros | 13 | 69 | 48 ± 9 | WHOI III: 4  WHO IV: 9 | Histology (*N*=13) | Based on histology with (predominant radiation necrosis with isolated atypical cells but no mitotic activity to suggest active tumour was classified as treatment effects. | 7∙2 (0∙5-18∙4) mo | 13 | 11 | 2 | 0 | TP/TrP: 22∙6 (6-136) mo |
| Kang et al., 2011 ^42^ | Retro | 35 | 49 | median 57  (23-82) | WHO III: 10  WHO IV: 25 | Imaging follow-up (*N*=35) | Progression was defined according to the MacDonald criteria. | 13∙2 (3∙3-58∙5) mo | 18 | 10 | 8 | 0 | TP: 3∙1 mo |
| Kim et al., 2010 ^43^ | Retro | 10 | 80 | 46 ± 11∙5  (31-66) | WHO III: 5  WHO IV: 5 | Histology (*N*=3), imaging follow-up (*N*=7) | Progression was defined according to the MacDonald criteria. | 22 mo | 10 | 6 | 4 | 0 | - |
| Kim et al., 2014 ^44^ | Retro | 169 | 47 | 52 (25-59) | WHO IV: 169 | Histology (*N*=87), imaging follow-up (*N*=82) | Pathologic features of TP included cellular sheets and/or nests of atypical cells, often with mitotic figures; fewer atypical cells, in a linear infiltrative configuration in parenchyma without prominent reactive changes. Pathologic features of TrP were geographic coagulative necrosis without pseudopalisading, vascular necrosis, vascular hyalinization, reactive vascular changes, dystrophic calcification, perivascular chronic inflammation, and gliosis with atypia.  Imaging based TP was progressive enhancement for >2 follow-up MRI, combined with clear neurologic deterioration that was attributable to enlarging contrast-enhancing lesions, causing treatment change. TrP was no change in treatment required, combined with decrease or stabilization of the enhancement for ≥6 mo during follow-up MRI scans. | - | 169 | 87 | 82 | 0 | TP: 10∙5 mo  TrP: 11∙9 mo postCCRT |
| Kong et al., 2009 (abstract) ^45^ | Pros | 76 | - | median 50 | WHO IV: 76 | - | - | - | 59 | 28 | 31 | 0 | TrP: *N*=18 during CCRT and *N*=13 during adjuvant TMZ |
| Kong et al., 2011 ^46^ | Pros | 90 | 54 | median 50  (25-74) | WHO IV: 90 | Imaging follow-up (*N*=90) | Progression was defined according to the MacDonald criteria. | 16∙5 (6∙2-48) mo | 59 | 33 | 26 | 0 | TP/TrP: <2 mo post CCRT |
| Larsen et al., 2013 ^47^ | Pros | 14 | 79 | 57 ± 10∙1  (38-76) | WHO III: 4  WHO IV: 10 | Histology (*N*=9), imaging follow-up (*N*=3); Death (*N*=2) | Progression was defined according to the MacDonald criteria. If a patient died prior to the completion of follow-up MRI due to circumstances clinically compatible with TP, they were classified as TP. | 3 mo | 14 | 11 | 2 | 1 | TP/TrP: 16 (3-48) mo postCCRT |
| Lee et al., 2012 ^48^ | Retro | 22 | 64 | 49 ± 15.8  (18-69) | WHO III: 3  WHO IV: 19 | Imaging follow-up (*N*=22) | Progression was defined according to the RANO criteria by two neuroradiologist in consensus. | 96 (80-102) d postCCRT | 22 | 10 | 12 | 0 | TP/TrP: <2 mo postCCRT |
| Linhares et al., 2013 ^49^ | Retro | 70 | 66 | median 62 (34-78) | WHO IV: 70 | Imaging follow-up (*N*=70) | Progression was defined according to the RANO criteria. | ≥ 3 mo post-CCRT | 15 | 13 | 2 | 0 | TP/TrP: 1 mo postCCRT |
| Matsuo et al., 2011 (abstract) ^50^ | - | 50 | - | - | WHO III: 14  WHO IV: 55 | Histology (*N*=50) | - | 20 mo | 50 | 34 | 16 | 0 | - |
| Matsusue et al., 2010 ^51^ | Retro | 6 | 50 | 50 ± 10∙7  (30-61) | WHO III: 1  WHO IV: 5 | Histology (*N*=1), imaging follow-up (*N*=5) | TP is enhancing lesion that increased in size on at least two serial MR examinations ≥2 mo. TrP were lesion that (1) disappeared or decreased in size on subsequent MR or (2) were present but unchanged in serial follow-up MR for ≥6 without receiving any further therapy. | - | 6 | 4 | 2 | 0 | TP: 12∙5 mo  TrP: 20 mo postCCRT |
| Melguizo et al., 2010 ^52^ | Retro | 295 | 58 | 58  (19-78) | WHO IV: 295 | Histology (*N*=34), imaging follow-up (*N*=261) | Solid areas of high grade tumour, occupying ≥70 % of the specimen was classified as TP. TrP included specimens which predominantly showed histologic features typically associated with treatment effect such as bland necrosis, fibrosis, gliosis, oedema, demyelination and vascular hyalinization. Additionally, concurrent presence of infiltrating atypical astrocytes or areas of tumour occupying <10 % of the specimen were included in this category.  Progression was defined as new or enlarging gadolinium enhancing lesion within 3 mo of completion of CCRT. TP was qualitatively defined as either increase in enhancement of a preexisting enhancing lesion or appearance of a new ‘‘solid or nodular’’ homogeneous or heterogeneous enhancing region either distinct from the primary site or along the margin of the surgical cavity. TrP was also qualitatively defined as areas of increased or new enhancement which had a reticulated or ‘‘soap bubbly’’ appearance. | - | 141 | 62 | 25 | 54 | TP/TrP: <3 mo postCCRT |
| Muggeri et al., 2010 (abstract) ^53^ | Retro | 61 | 47 | median 60  (18-72) | WHO IV: 61 | Imaging follow-up (*N*=61) | MRI not otherwise specified | 12 mo | 35 | 14 | 21 | 0 | TP/TrP: 1 mo postCCRT |
| Nakajima et al., 2009 ^54^ | Retro | 14 | 71 | 45 ± 16∙7 | WHO III: 6 WHO IV: 8 | Histology (*N*=11), imaging follow-up (*N*=3) | - | 66∙8 mo | 14 | 5 | 9 | 0 | - |
| Nasseri et al., 2014 ^55^ | Retro | 56 | 64 | 55 ± 13∙8 | WHO IV: 56 | Imaging follow-up (*N*=56) | Progression was based upon the RANO criteria. | 459 d | 48 | 21 | 27 | 0 | TrP: *N*=19 <3 mo, *N*=8 >3 mo postCCRT |
| Neal et al., 2013 ^56^ | Pros | 63 | 56 | median 55  19-76 | WHO IV: 63 | Imaging follow-up (*N*=63) | TrP was defined in those patients with early changes in  enhancement consistent with progression that showed subsequent radiographic stabilization or improvement. | - | 24 | 12 | 12 | 0 | TP/TrP: <180 days postCCRT |
| Palumbo et al., 2006 ^82^ | Pros | 24 | 73* | 53 ± 13∙7  (25-76) | WHO III: 8  WHO IV: 16 | Histology (*N*=24) | - | 3-12 mo | 24 | 17 | 7 | 0 | - |
| Peca et al., 2009 ^57^ | Pros | 50 | 45 | 53  (28-72) | WHO IV: 50 | Histology (*N*=10), imaging follow-up (*N*=1) | - | 16-50 mo | 15 | 7 | 4 | 4 | TP/TrP: 6 mo postCCRT |
| Pica et al., 2012 (abstract) ^58^ | Pros | 26 | - | - | WHO III:10 WHO IV: 16 | Histology (*N*=8), not described (*N*=18) | - | 6∙5 mo | 26 | 11 | 15 | 0 | - |
| Pouleau et al., 2012 ^59^ | Retro | 63 | 63 | median 60  (27-78) | WHO IV: 63 | Imaging follow-up (*N*=63) | TrP is progressive enhancing lesions with peritumoural oedema at MRI ≤8 wk postCCRT, without clinical signs of deterioration, that stabilizes or even resolves after additional cycles of adjuvant TMZ. | - | 33 | 26 | 7 | 0 | TP/TrP: <8 weeks post CCRT |
| Radbruch et al., 2013 (abstract) ^60^ | Retro | 112 | - | - | WHO IV: 112 | Imaging follow-up (*N*=112) | According to the RANO criteria | 12 mo post surgery | 85 | 78 | 7 | 0 | - |
| Radbruch et al., 2015 ^61^ | Retro | 79 | - | - | WHO IV: 79 | Imaging follow-up (*N*=79) | Progression is ≥25% increase of enhancement of an original lesion with ≥10 mm of perpendicular diameters or a new nodular component ≥10 mm within the radiation field in the first, second, third, or fourth follow-up compared with the baseline examination. | 12 mo | 79 | 70 | 9 | 0 | TP: 87% postCCRT  TrP: 67% postCCRT |
| Reddy et al., 2013 ^62^ | Retro | 51 | 65 | 47  (22-71) | WHO III: 16 WHO IV: 35 | Histology (*N*=51) | An experienced neuropathologist used the hematoxylin and eosin staining. TP was classified if the tissue specimens contained ≥70% recurrent tumour. By contrast, if ≥70% necrotic tissue was demonstrated, they were characterised as trP. | - | 51 | 20 | 18 | 13 | TP/TrP: 7∙3 mo postCCRT |
| Roldán et al., 2009 ^63^ | Retro | 43 | 58 | median 55  (29-71) | WHO IV: 43 | Imaging follow-up (*N=*43) | Progression is >25% increase enhancement or a new enhancing lesion. TP was defined as further deterioration on the MRI done after receiving at least one cycle of adjuvant TMZ. TrP was defined as increased enhancement which either stabilized or resolved on the MRI post TMZ. | - | 25 | 10 | 10 | 5 | TP/TrP: post CCRT |
| Sanghera et al., 2010 ^64^ | Pros | 111 | 63 | median 58 | WHO III: 7  WHO IV: 104 | Imaging follow-up (*N=*104) | Contrast enhanced MR or CT scans were used to define response. TP is further radiological progression or death ≤6 mo postCCRT. TrP is no further radiological progression, without using salvage therapy other than the monthly adjuvant TMZ with stable dexamethasone dose during the same 6 mo. | 55 wk post CCRT | 25 | 15 | 7 | 5 | TP/TrP: <8 weeks postRT |
| Seeger et al., 2013 ^65^ | Retro | 40 | 60 | 54 ± 13∙6 | WHO III+IV: 40 | Imaging follow-up (*N*=40) | Progression was classified according to the RANO criteria | 10 (6-15) mo in  trP pa-tients | 40 | 23 | 17 | 0 | - |
| Shah et al., 2009 (abstract) ^66^ | Retro | 39 | 79 | 54 | WHO IV: 39 | Histology (*N*=8), imaging follow-up (*N*=31) | TP was classified if the abnormalities continued to worsen and trP if the abnormalities improved or stabilized. | 6 mo post-CCRT | 39 | 16 | 23 | 0 | TP/TrP: 0 mo postRT |
| Song et al., 2013 ^67^ | Retro | 20 | 50 | 51 ± 13∙5 | WHO IV: 20 | Imaging follow-up (*N*=20) | Progression was classified according to the RANO criteria | - | 20 | 10 | 10 | 0 | TP/TrP: Post CCRT |
| Suh et al., 2013 ^68^ | Retro | 79 | 46 | 51  (25-69) | WHOI IV: 79 | Histology (*N*=24), imaging follow-up (*N*=55) | TP was diagnosed if a steady increase in enhancement on ≥2 follow-up MRI with an interval of 2-3 mo, and clear clinical deterioration that was not attributable to concurrent medication or comorbid conditions, prompted a change in treatment. TrP was defined as no change in treatment was required for a ≥6 mo post CCRT. | - | 79 | 42 | 37 | 0 | TP/TrP: 4-5 wk post CCRT |
| Sundgren et al., 2006 ^83^ | Retro | 13 | 54 | 46  (31-61) | WHO III: 9  WHO IV: 4 | Histology (*N*=5, imaging follow-up (*N*=8) | TrP is long-term clinical stability or clinical improvement, and lesion stability or regression based on imaging, while progression in lesion size and clinical deterioration or death implied TP. | 19∙4 mo post-CCRT | 13 | 7 | 6 | 0 | TP: 37∙6 mo  TrP: 14∙8 mo postCCRT |
| Taal et al., 2008 ^69^ | Retro | 85 | 66 | median 50  (18-68) | WHO III: 17  WHO IV: 68 | Histology (*N*=1), imaging follow-up (*N*=84) | Progression was defined as 25% increase noted on the MRI scan 4 weeks postCCRT with or without neurologic  deterioration, and on a stable or higher dose of dexamethasone. TP was scored if the patient developed additional progression ≤6 mo. TrP was scored if the patients 1) had at least a 50% decrease in the enhancing lesion during further follow-up, while remaining neurologically stable and on a stable or decreasing dose of dexamethasone (a ‘partial response’ according to the criteria of Macdonald) or 2) remained clinically and radiologically stable with a stable or decreased dosage of steroids for ≥6 mo postCCRT without any further treatment other than adjuvant cycles of TMZ. | - | 36 | 18 | 18 | 0 | TP/TrP: 4 wk postCCRT |
| Tie et al., 2008 ^70^ | Retro | 19 | 71 | 51  (25-78) | WHO III: 12  WHO IV:7 | Histology (*N*=9), imaging follow-up (*N*=10) | TP and trP was based upon histology or subsequent clinical and/or radiological findings. | 33 mo | 19 | 16 | 3 | 0 | - |
| Tiwari et al., 2013 (abstract) ^71^ | Retro | 17 | - | - | WHO IV: 17 | Histology (*N=*17) | - | - | 17 | 7 | 10 | 0 | - |
| Topkan et al., 2012 ^72^ | Retro | 63 | 70 | median 58  (29-69) | WHO IV: 63 | Histology (*N*=28) | Progression was based upon T1C within 6 mo from the last day of CCRT, not otherwise specified. Histology was acquired in all patients with early radiologic progression. To confirm histological trP supportive MIB-1findings were mandatory. | 19∙7 mo | 28 | 16 | 12 | 0 | TrP: 2∙9 mo postCCRT |
| Tsien et al., 2010 ^73^ | Pros | 27 | - | 52 ± 3∙1 | WHO III: 4 WHO IV: 23 | Imaging (*N*=27) | Progression was ≥4 mL of enhancing lesion. MacDonald criteria were used to determine TP and trP | - | 27 | 15 | 12 | 0 | TP/TrP: <3 mo |
| Vafiadis et al., 2010 (abstract) ^74^ | - | 15 | - | - | WHO III + IV: 15 | Histology (*N*=7), imaging follow-up (*N*=8) | Increasing lesions and clinical worsening was considered TP. Decreasing or disappearing lesions was regarded as trP. | - | 15 | 9 | 6 | 0 | - |
| van Mieghem et al., 2013 ^75^ | Retro | 67 | 64 | median 57  (26-78) | WHO IV: 57 | Imaging follow-up (*N*=67) | Progression was defined as increase in tumour or appearance of new lesions ≤3 mo postCCRT. TP was ≥25% enhancement that increased further. TrP was ≥25% enhancement that stabilized. | - | 40 | 22 | 18 | 0 | - |
| Wertz et al., 2012 (abstract) ^76^ | Retro | 58 | - | - | WHO IV: 58 | Imaging follow-up (*N*=58) | Progression is progression on the MRI 4 wk postCCRT according to the RANO criteria. TP showed further progression on the 3 and 5 mo MRI postCCRT. TrP showed stable MRI on the 3 and 5 mo postCCRT. | 5 mo postRT | 29 | 19 | 10 | 0 | - |
| Yaman et al., 2010 ^77^ | Retro | 67 | 51 | 45  (23-74) | WHO III: 9  WHO IV: 58 | Histology (N=3), imaging follow-up (N=14) | Progression was increased enhancement, mass effect, oedema or high signal on T2 and FLAIR. TP and trP were differentiated with MRS not otherwise specified and trP was confirmed with histology in 75%. | 19 mo | 17 | 13 | 4 | 0 | - |
| Young et al., 2011 ^78^ | Retro | 91 | 38 | 58  (21-84) | WHO IV: 90 | Histology (*N*=28), imaging follow-up (*N*=65) | Progression is new or increased (≥25%) enhancing  lesion. TP was ≥25% enhancement and/or clinical  deterioration that required a change in treatment ≤6  mo postCCRT. TrP was progression not classified as TP. | Full clinical file | 91 | 61 | 29 | 1 | TP/TrP: 2-4 wk postCCRT |
| Zeng et al., 2007 ^84^ | Retro | 28 | 64 | 40 ± 9∙8  (23-65) | WHO III: 20  WHO IV: 8 | Histology (*N*=21), imaging follow-up (*N*=7) | TP histopathology showed active tumour or follow-up MRI showing mass effect and steady growth of enhancement. TrP was verification of radiation injury without tumour histologically or stable-appearing or resolving enhancement on subsequent MRI. | - | 28 | 19 | 9 | 0 | - |

Characteristics of 73 included studies. Abbreviations: AMT = α-methyl-L-tryptophan; C = carbon; CCRT = concomitant chemoradiotherapy; CT = computed tomography; d = days; DCE = dynamic contrast-enhanced perfusion; DMSA = dimercaptosuccinic acid; DSC = dynamic susceptibility contrast perfusion; DTI = diffusion tensor imaging; DWI = diffusion weighted imaging; FLAIR = fluid attenuated inversion recovery; IQR = interquartile range; MET = methionine; Mo = months; MRI = magnetic resonance maging; MRS = magnetic resonance spectroscopy; N = number; PET = positron emission tomography; Pros = prospective; PWI = perfusion weighted imaging; Retro = retrospective; SD = standard deviation; SPECT = single positron emission computed tomography; SWI = susceptibility weighted imaging; T = Tesla; T1C = T1 post contrast; Tc=technetium; TP = tumour progression; trP = treatment induced progression; WHO = World Health Organisation; wk = weeks; y = years;
